# Deciphering acoustic emission signals in drought stressed branches: the missing link between source and sensor

**DOI:** 10.3389/fpls.2015.00494

**Published:** 2015-07-02

**Authors:** Lidewei L. Vergeynst, Markus G. R. Sause, Marvin A. Hamstad, Kathy Steppe

**Affiliations:** ^1^Laboratory of Plant Ecology, Department of Applied Ecology and Environmental Biology, Faculty of Bioscience Engineering, Ghent UniversityGhent, Belgium; ^2^Experimental Physics II, Institute of Physics, University of AugsburgAugsburg, Germany; ^3^Department of Mechanical and Materials Engineering, University of DenverDenver, CO, USA

**Keywords:** acoustic emission detection, cavitation, drought, finite-element modeling, point-contact sensor, waveform analysis

## Abstract

When drought occurs in plants, acoustic emission (AE) signals can be detected, but the actual causes of these signals are still unknown. By analyzing the waveforms of the measured signals, it should, however, be possible to trace the characteristics of the AE source and get information about the underlying physiological processes. A problem encountered during this analysis is that the waveform changes significantly from source to sensor and lack of knowledge on wave propagation impedes research progress made in this field. We used finite element modeling and the well-known pencil lead break source to investigate wave propagation in a branch. A cylindrical rod of polyvinyl chloride was first used to identify the theoretical propagation modes. Two wave propagation modes could be distinguished and we used the finite element model to interpret their behavior in terms of source position for both the PVC rod and a wooden rod. Both wave propagation modes were also identified in drying-induced signals from woody branches, and we used the obtained insights to provide recommendations for further AE research in plant science.

## Introduction

For almost 50 years, scientists have been studying the sounds emitted by plant organs suffering from drought. The sources of the acoustic emissions (AEs) have been under debate ever since, which complicates the interpretation of AE data in terms of plant physiological processes. Recently, Ponomarenko et al. (2014) demonstrated that nucleation of an air bubble inside a xylem conduit in a microscopic slice of conifer xylem can produce a detectable AE signal. However, other types of AE sources must exist in plants to explain the AE signals observed during dehydration coming from other tissues in addition to xylem (Milburn, 1973a; Kikuta, 2003), during drying of wood beyond the point when all xylem conduits are empty (Vergeynst et al., 2015), during re-watering (Milburn, 1973b), and also during freezing (Raschi et al., 1989; Mayr and Sperry, 2010; Charrier et al., 2014) and thawing (Raschi et al., 1989; Mayr and Sperry, 2010). For small samples, it is possible to trace the AE source by simultaneous microscopic visualization at very high temporal resolution with multiple frames per second (Ponomarenko et al., 2014). However, when measuring on larger samples or actual plants, this approach is not feasible because the current visualization techniques do not enable combination of high spatial and temporal resolution on the scale of a macroscopic sample. Another method to trace the source of AE signals would be to extract the information that is included in the detected AE signals by analyzing the waveforms. Attempts have been made to interpret the waveforms (Milburn and Johnson, 1966; Tyree and Dixon, 1983; Laschimke et al., 2006; Rosner et al., 2006), but to date they remain indecisive about the actual AE sources. We believe that the major hurdle for interpreting AE signals is the general lack of knowledge about wave propagation in the measured plant organ, since the wave propagation has significant impact on the detected AE signals.

The choice of the AE sensor is also very important to properly understand and interpret the wave propagation features. Previous attempts to interpret whole waveforms were limited by the use of resonant sensors (Laschimke et al., 2006) or broadband sensors with non-flat frequency response (Tyree and Dixon, 1983). These sensors strongly influence the frequency spectrum and the time domain of the detected AE signal. Moreover, common AE sensors are cylindrically shaped so that the captured signal is the average response over the disk-shaped sensitive element. The detection of waves that do not arrive normal to the sensor face will be distorted by the large area of the sensitive element (so-called aperture effect), especially affecting frequencies where the wavelengths are smaller than the sensor diameter (McLaskey and Glaser, 2012). The aperture effect can be minimized by the use of point-contact sensors of which the diameter of the sensitive element is much smaller than the signal wavelengths (Sause et al., 2012a). Finally, it is important to know the directivity of the sensor and the type of response. The point-contact sensor used in this study is only sensitive to out-of-plane displacements, perpendicular to the sensor face. Other sensors can have sensitivity to combinations of in- or out-of-plane displacement, velocity, and acceleration (Ono et al., 2008; McLaskey and Glaser, 2012). Such mixed sensitivity complicates signal interpretation.

In this study, we focus on wave propagation in woody branches. During propagation of acoustic waves through the branch, processes such as material attenuation, scattering, geometrical attenuation, and dispersion change the waves and typically decrease the wave amplitude when it travels away from the AE source location (Bucur and Böhnke, 1994). Moreover, the majority of the signals have been reflected several times at the branch surface before they reach the AE detector. These reflections result in a reduced geometrical attenuation, which allows the waves to travel and be detected at longer distances than in an infinite medium. In addition, the interactions of multiple reflections by the geometric structure result in so-called guided waves (Rose, 2004). The fact that the branch acts as a waveguide strongly affects signal interpretation, but so far this has not been taken into account in the literature that we have found for AE studies in plants. The objectives of this research are twofold. First, we aim at understanding how acoustic waves are propagated in a branch with rod-type geometry and how source location may affect the composition of the detected waveform. Second, we use these insights to explain waveform features observed in drying-induced AE signals from desiccating branches and we provide a solid framework for further AE research in plant science.

## Materials and Methods

### Pencil Lead Break as Artificial AE Source

In order to examine wave propagation and effects of source location, we used the well-known pencil lead break (PLB) source. The breakage of a pencil lead, according to ASTM Standard E976, is extensively used as an artificial source in AE technology (Tyree and Sperry, 1989; Berthelot et al., 1992; Hamstad et al., 1994; Sause, 2011; Dehghan Niri and Salamone, 2012; Strantza et al., 2014). When pressing the lead of a propelling pencil firmly against the surface, the lead will break and local stresses in the surface will be released so that the surface jumps back to its original position. This unloading force as a function of time *F(t)* (N) can be described by a cosine bell function (Eq. 1; Hamstad et al., 1999; Sause, 2011), which is directed normal to the surface.

(1) $$\begin{array}{c} F(t)=0\;\;\text{for}\;\text{t}\;<\;0\\ F(t)=0.5-0.5\text{cos}\frac{\text{π}t}{\tau }\;\text{for}0\;\leq \;\text{t}\;\leq \;\text{τ }\\ F(t)=1\text{ for}\;\text{t}\;>\;\text{τ} \end{array}$$

The rise time *τ* is dependent on the elastic properties of the surface material. This function is suitable to simulate the acoustic wave caused by a PLB (Sause, 2011) in the far field. For a PLB on aluminum, the rise time was found to be 1 μs (Sause, 2011) and we use this value as a reasonable estimate for the rise time on a PVC surface. The far field refers to distances from the AE source at which the signals have been significantly changed by the specimen geometry (Hamstad et al., 1994) and guided waves can be observed. In a PVC rod, guided waves are already developed at a propagation distance of three times the rod diameter (Marvin Hamstad, personal communication).

We investigated the direct pressure wave (without reflections) that is created by a PLB (2H, 0.3 mm, Pentel) directly on the sensor tip (KRNBB-PC sensor, KRN Services, Richland, WA, USA) and on a wooden board across from the sensor tip. For the latter, a sensor was installed in the middle of a board of dry Mahogany wood with dimensions 439 mm × 122 mm × 19.6 mm and the PLB was made on the opposite side of the board, directly across from the sensor tip. Another PLB was carried out at 2 cm away from the center of the sensor.

### Finite Element Modeling to Investigate Wave Propagation

**FIGURE 1:**
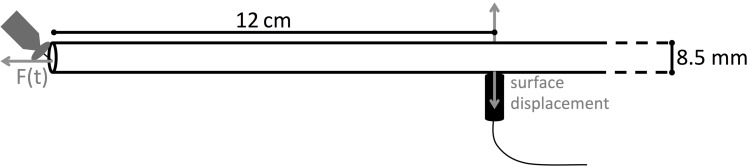
**Schematic representation of the modeled PVC and wooden rod.** Surface normal displacement is evaluated at 12 cm from the rod end, where a pencil lead break (PLB) is made.

Wave propagation is a micro-mechanical process and can be seen as a series of stretching and relaxation of springs inside the material. We simulated wave propagation with finite element modeling of a linear elastic material, using the software COMSOL Multiphysics. First, a PVC rod was used for investigation of the wave modes because the simple geometry (8.5 mm diameter, 1 m length) and isotropic elastic properties facilitated the calculation of group velocity curves (explained below). We modeled the PVC rod as an isotropic cylinder with density 1500 kg m^-3^, modulus of elasticity equal to 4.693 GPa and a Poisson’s ratio of 0.3664 (Kaye & Laby Online, 2005). For modeling of a wooden rod, we used the elastic constants of ash (**Table 1**) from Dinckal (2011) and a fresh wood density of 1000 kg m^-3^. The selected model was convergent with models of a higher resolution when applying a time step of 0.1 μs and a maximum mesh size of 1 mm. To enhance representation of fine details, mesh refining down to 0.01 mm was allowed with a maximum element growth rate of 1.5 per element. The far-field waveforms were simulated as displacements normal to the surface at 12 cm from the cylinder end (**Figure 1**), where the PLB was simulated. The force exerted by the simulated PLB was implemented as a cosine bell function (Eq. 1), acting normal to the rod end. The surface displacements, which could be calculated at each point on the rod surface, represent the waveforms that would be detected by a broadband point-contact sensor with a near flat with frequency displacement response (Hamstad, 1997; McLaskey and Glaser, 2012; Sause et al., 2012a). We examined the waveforms with respect to AE source location by first varying the distance from the PLB location to the center of the rod (0–3 mm off center). A second examination varied the angle (0–90°) between a radius to the PLB location and a radius to the detector location viewed from the end of the rod.

**Table 1:** Stiffness tensor (C, Voigt notation) with elastic constants (C_ij_, MPa) of ash from Dinckal (2011), used for modeling the wooden rod.

C_11_ = 2.439	C_12_ = 1.037	C_13_ = 1.968	C_14_ = 0	C_15_ = 0	C_16_ = 0
C_21_ = 1.037	C_22_ = 1.439	C_23_ = 1.485	C_24_ = 0	C_25_ = 0	C_26_ = 0
C_31_ = 1.968	C_32_ = 1.485	C_33_ = 17.000	C_34_ = 0	C_35_ = 0	C_36_ = 0
C_41_ = 0	C_42_ = 0	C_43_ = 0	C_44_ = 1.218	C_45_ = 0	C_46_ = 0
C_51_ = 0	C_52_ = 0	C_53_ = 0	C_54_ = 0	C_55_ = 1.720	C_56_ = 0
C_61_ = 0	C_62_ = 0	C_63_ = 0	C_64_ = 0	C_65_ = 0	C_66_ = 0.500

*A cylindrical coordinate system was used with the first, second, and third axis being aligned with the radial, tangential, and longitudinal direction, respectively, of the internal wood structure*.

### Verification of the Model

Although the finite element method has been shown to be valid for calculating guided wave propagation (Sause et al., 2013), we verified the integrity of our model by comparison with an experimental PLB. We broke a pencil lead (2H, 0.3 mm, Pentel) of ∼3 mm length on the end of the PVC rod at 1 mm from the rod axis toward the side of the sensor location (**Figure 1**). We used a broadband point-contact sensor (KRNBB-PC sensor) that is sensitive to surface displacements in the frequency range 20–1000 kHz (Glaser and Nelson, 1992; McLaskey and Glaser, 2012). These sensors with conical piezoelectric elements have a very flat spectral response (Sause et al., 2012a), which makes them most appropriate to experimentally verify the simulated signals. For detailed comparison with the simulations, the experimental signals were collected at much higher sample rate than usually required for AE monitoring in wood. Signals with a length of 10240 samples were collected at 10 M samples s^-1^ with 250 μs pretrigger time. Both the simulated and the experimental signals were filtered with 7–100 kHz band-pass. We used fourth order Butterworth filters (Ligges et al., 2015) for the 100 kHz low-pass filtering of both signals, since a valid comparison can only be made for both signals having the same frequency range. For the 7 kHz filtering, the experimental signal was filtered electronically, and for the simulated signal a fourth order Butterworth filter was used. The 100 kHz low-pass was necessary because we observed that as the frequencies increased above ∼100 kHz they were increasingly attenuated in the experimental signals at 12 cm source-sensor distance, while material attenuation was not included in the model. We assumed that material attenuation was low below 100 kHz. Based on the very good match of the model versus the experimental signals (see later section), we concluded that this was a reasonable assumption. We applied a high-pass filter of 7 kHz because this was the lower limit of the frequency range of the preamplifier (model AMP-1BB-J, KRN Services, Richland, WA, USA).

### Identification of the Guided Wave Modes

The presence of different guided wave modes was investigated using the Choi–Williams distribution (CWD) of the simulated waveform (Hamstad, 2008), which is a spectrogram showing the wave intensity with a color or varied black intensity scale on a plot of frequency versus time for the signal duration. This was carried out with the AGU-Vallen Wavelet software (www.vallen.de/downloads) using a frequency resolution of 1.221 kHz, 112 terms in the damping summation and an exponential damping parameter of 20. For the CWD representation we filtered the simulated signal with a 20–500 kHz band-pass in order to better visualize frequencies up to 500 kHz, which were otherwise overwhelmed in the CWD diagram by the high intensities at low frequencies.

All guided wave modes in a rod with a certain diameter, known density, and elastic properties can be characterized by so-called dispersion curves (Rose, 2004), which describe the relation between frequency and velocity. The group velocity at a certain frequency can be understood as the propagation velocity of wave packets containing frequencies in a narrow band around this value. Group velocity dispersion curves were calculated for the PVC rod at a propagation distance of 12 cm, velocities were converted into arrival time and the curves were superimposed on the plot of the CWD for comparison and identification of the wave modes. For the calculation of dispersion curves for cylindrical wave guides, an open-source Matlab program called PCDISP (Seco and Jiménez, 2012) is available.

### Drying-Induced AE Signals

Excised branches of grapevine (*Vitis vinifera*), ash (*Fraxinus excelsior*), ivy (*Hedera helix*), poplar (*Populus × canadensis*), and common beech (*Fagus sylvatica*) with diameters of ∼1 cm and ∼1 m length were monitored with the broadband point-contact sensors during bench dehydration, without removing leaves or bark. On the branch of ash and grapevine we installed a pair of sensors opposite each other, while on the other branches only one sensor was installed. Vacuum grease (High-Vacuum Grease, Dow Corning, Seneffe, Belgium) was applied between wood and sensor tip to ensure good acoustic transmission by removing air pockets between the rough surface and the sensor face (Miller, 1987). Signals with a length of 4096 samples that crossed the threshold of 27 dB_AE_ were collected in the frequency range 20–1000 kHz at 5 M samples s^-1^ and with 250 μs pretrigger time. The signal was amplified by an internal JFET amplifier inside the sensor (20 dB) and an in-line preamplifier (35.6 dB).

## Results

### Pencil Lead Break

**FIGURE 2:**
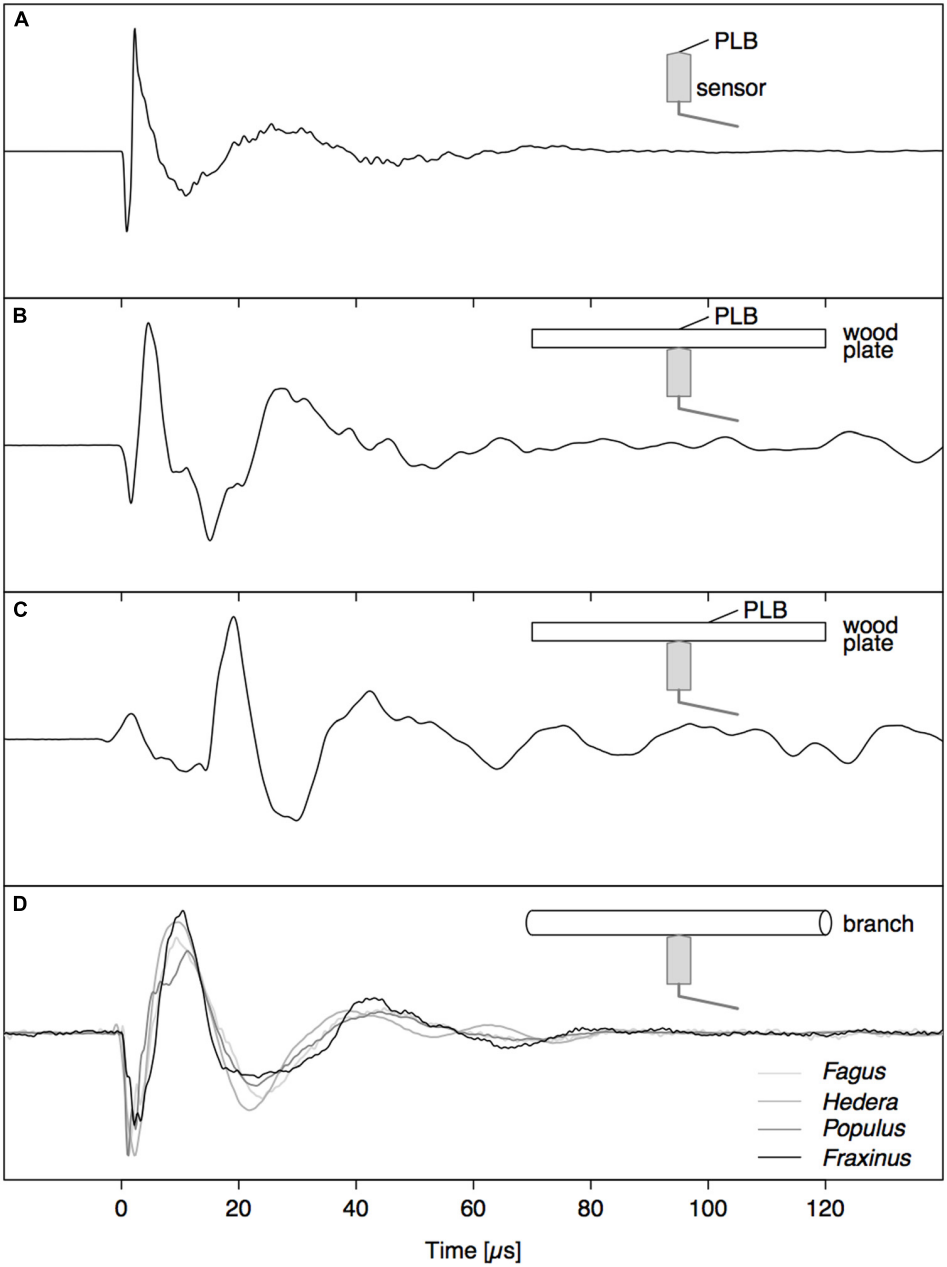
**Comparison between signals caused by a PLB (A–C) and nearby drying-induced AE signals from dehydrating branches (D). (A)** PLB on sensor tip, **(B)** PLB on wood directly opposite to the sensor, and **(C)** signal from PLB on the opposite side of the wood at 2 cm from sensor location.

A PLB directly on the sensor tip (**Figure 2A**) resulted in a waveform with two sharp peaks, a fast loading (negative peak) and unloading (positive peak) of the sensor tip, followed by a damped oscillation. The waveform generated by a PLB on wood across from the sensor (**Figure 2B**) also featured the initial two peaks, but the dynamics were slower (broader peaks). These differences are due to the more than ten times higher modulus of elasticity of the nickel faceplate of the sensor compared to wood, and also the strong attenuation of high frequencies on the path through the wood. Also, a reflection of the wave from the opposite surface of the wood board arrived after about 10 μs, which distorted the damped oscillation. When the pencil lead was broken 2 cm away from the sensor tip on the opposite side of the wood board (**Figure 2C**), the initial part of the waveform changed substantially, which illustrates the need for understanding wave propagation before interpreting the detected signals.

### Guided Waves

**FIGURE 3:**
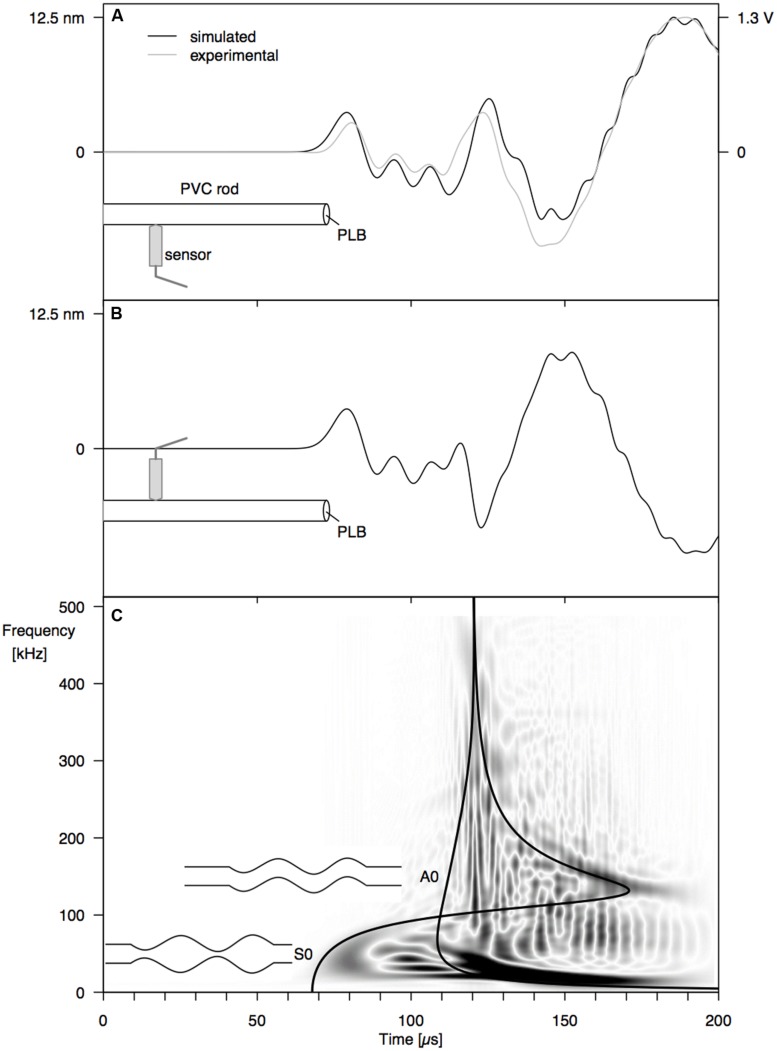
**Surface displacement at 12 cm from the end of a PVC rod, resulting from a PLB at the rod end, at 1 mm off center: (A) simulated (black) and experimental (gray) waveforms at 0° from the PLB location, (B) simulated waveform at 180°, and (C) Choi–Williams transformation of the simulated signal in (A) with velocity dispersion curves for the S0 and A0 mode**.

Good correspondence between simulated surface displacement and experimentally obtained waveform at 12 cm from the PLB at the end of the PVC rod (**Figure 1**) confirms that the model configuration was valid (**Figure 3A**). Comparing simulated signals on both sides of the rod (**Figures 3A,B**), we can identify a first arrival of a symmetric wave at around 70 μs, due to the simultaneous out- and inward displacement at opposite sides of the rod. Subsequently, an anti-symmetric wave with lower velocity arrives at around 110 μs, with outward displacement at one side of the rod occurring simultaneously with inward displacement at the opposite side. A schematic representation of both wave modes is shown in **Figure 3c**. From the velocity dispersion curves of a cylindrical rod (**Figure 3C**) it can be seen that these arrival times correspond to the arrival of the fundamental symmetric (S0) and anti-symmetric (A0) wave mode, respectively. The good correspondence between CWD and the dispersion curves confirms that we are dealing with the S0 and A0 mode and provides added validation of the correctness of the finite element model.

### Influence of Source Location

**FIGURE 4:**
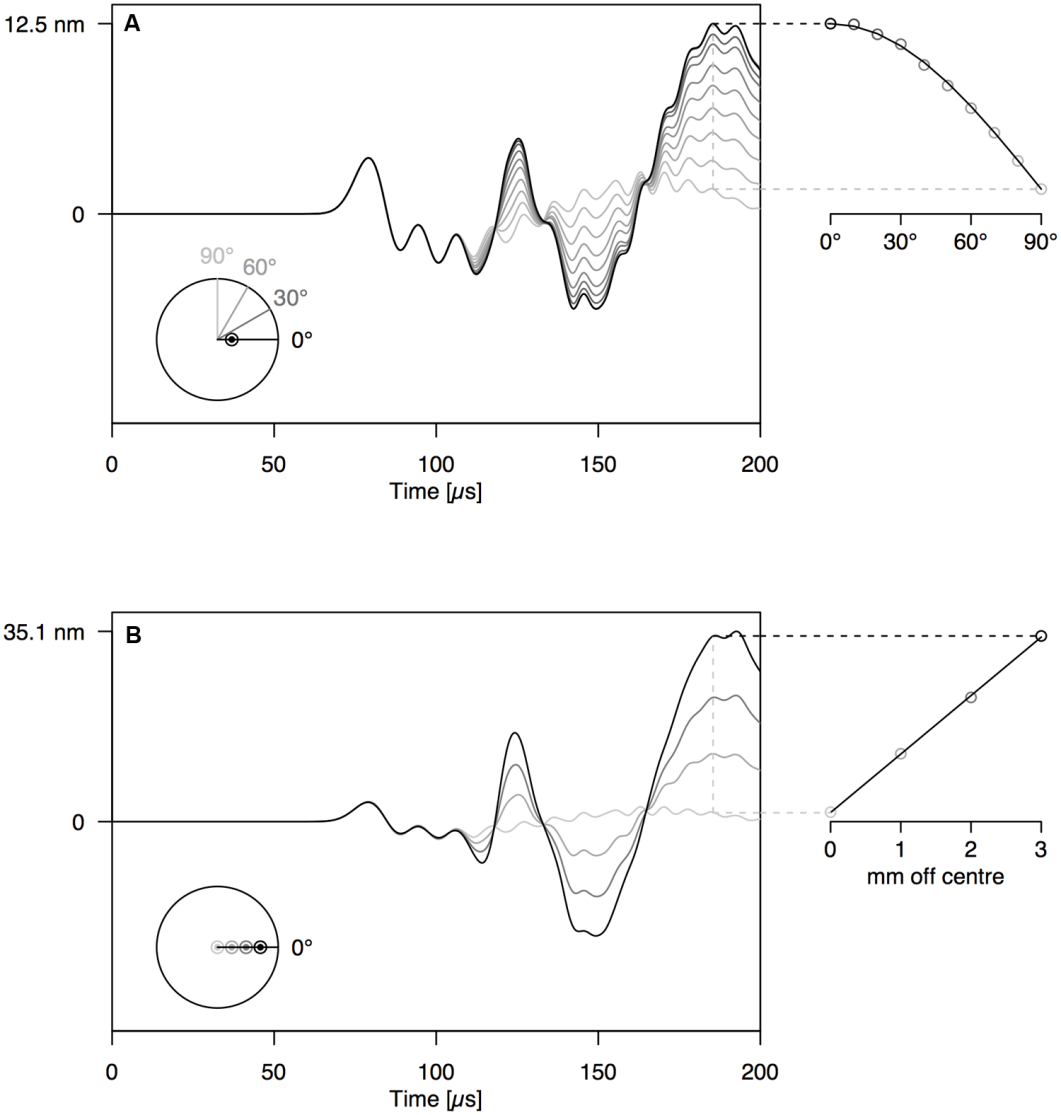
**(A)** Waveforms simulated at 12 cm from the end of the PVC rod at an angle of 0–90° with the location of the PLB, which was made at 1 mm off center on the rod end. **(B)** Waveforms simulated at 12 cm from the end of the PVC rod, with PLB at 0–3 mm from the center of the rod end. The schematic at the bottom-left of the graphs shows the location of sources and sensors, when viewed perpendicular to the rod end.

**FIGURE 5:**
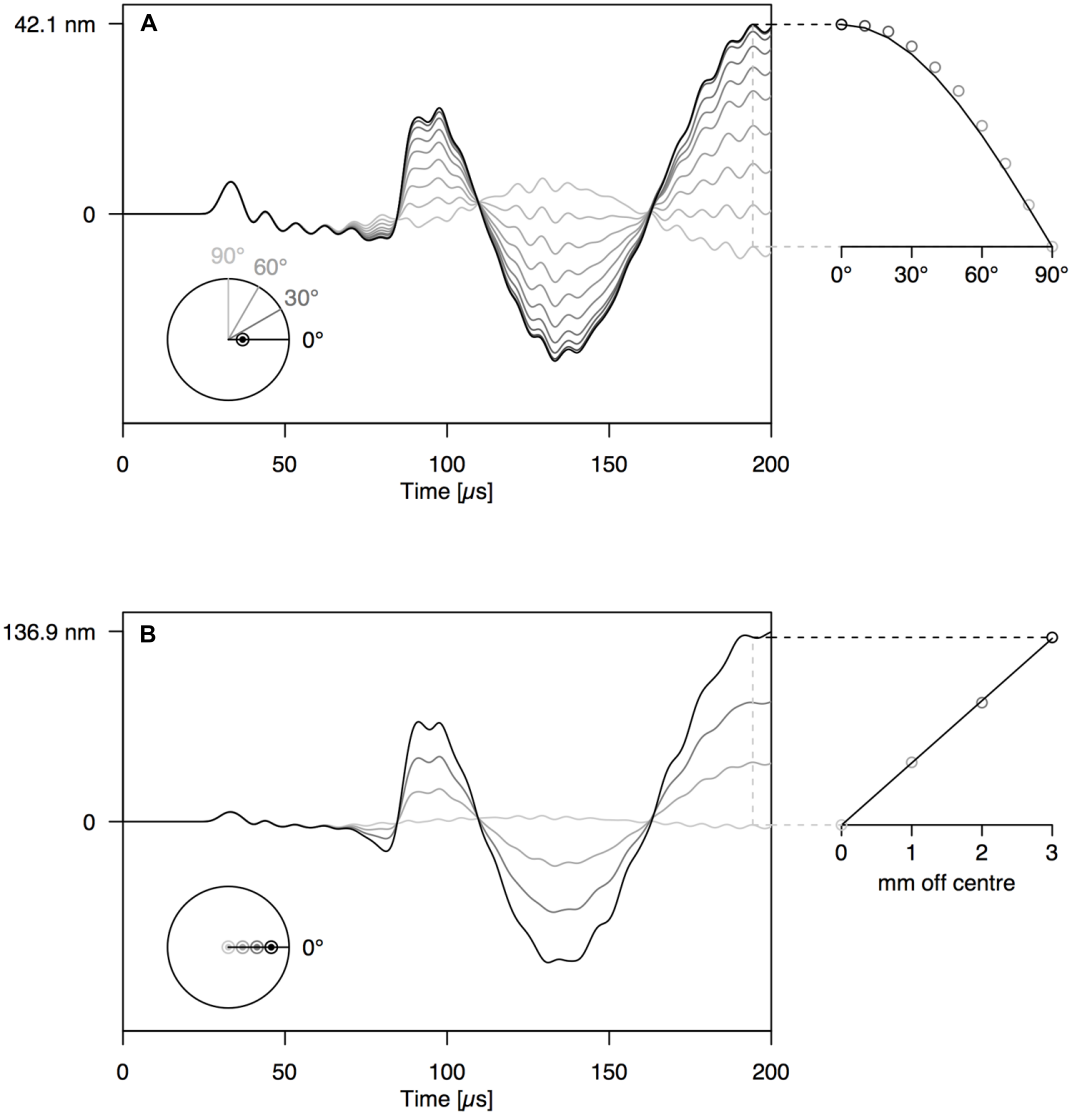
**Same set-up as **Figure 4** on anisotropic wooden rod shows similar behavior of A- and S-modes**.

When increasing the angle between the AE source (PLB) and the sensor, the amplitude of the first-arriving S0 mode was not affected (**Figures 4A and 5A**). The A0 mode amplitude, in contrast, decreased with increasing detection angle, until it completely disappeared at 90°. The A0 amplitude decrease was proportionate to the cosine of the detector angle, but because of the anisotropy of wood, the decrease in A0 amplitude deviated somewhat from the cosine function for the wooden rod (**Figure 5A**). The distance of the AE source from the axis of the rod has a significant influence on the amplitude of the A0 mode as well (**Figures 4B and 5B**). With constant angle between source and detector (0°), the A0 mode amplitude decreased linearly with decreasing distance between the source and the center of the rod.

### Drying-Induced AEs

**FIGURE 6:**
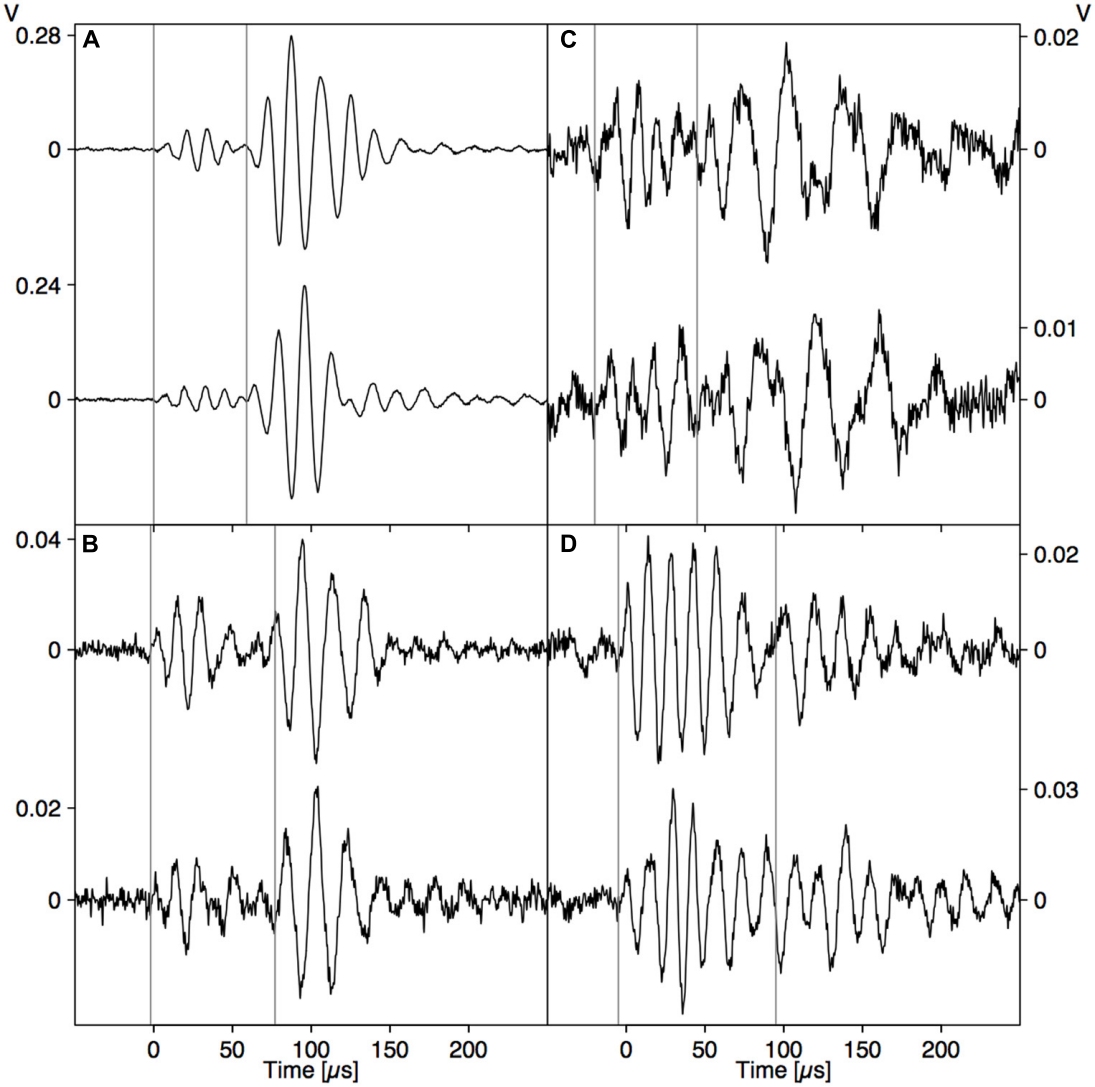
**Two examples of AE events detected simultaneously by two sensors installed opposite each other on dehydrating branches of grapevine (*Vitis vinifera*; A,B) and ash (*Fraxinus excelsior*; C,D).** The vertical lines indicate the arrival of the symmetrical (first line) and anti-symmetrical (second line) wave modes.

In the whole range of AE signals collected from dehydrating branches, we selected some typical near- and far-field signals based on characteristic AE features. The near-field signals (**Figure 2D**) were characterized by a small time to reach the peak amplitude and fast decay. The far-field signals (**Figure 6**) did not feature the initial sharp peaks, but were smeared in time and were attenuated due to the propagation along the branch. The waveforms of the far-field signals could be divided into two parts based on the signals detected at both sides of the branch. At the beginning of the waveforms (first vertical line), both signals were symmetric, with positive peaks co-occurring at the same times on opposite sides of the branch. After some time (second vertical line) both signals became anti-symmetric, with positive peaks co-occurring with negative peaks at the same times on the opposite side of the branch. The difference in arrival time between the symmetric and anti-symmetric wave modes was larger for signal (B) than (A), two examples from a grapevine branch, and larger for signal (D) than (C), two examples from an ash branch. This suggests that for the signals on grapevine, the AE source was located closer to the sensor set (A) and likewise closer to sensor set (C) for the ash examples.

## Discussion

### Behavior of Guided Waves in a Branch

Acoustic emission signal analysis requires a thorough understanding of wave propagation from source to sensor. When a displacement wave propagates along a waveguide, the shape of the initial displacement wave (**Figure 2B**) may become distorted beyond recognition (**Figures 2C and 3**). However, this distortion is not random and after propagating some distance away from the source, guided wave modes are developed. The finite element model, which was proven to simulate far-field AE signals correctly (**Figure 3**), was used to investigate wave propagation between source and sensor. The basic principles of guided wave modes that were investigated on the isotropic PVC rod also applied on the wooden rod (**Figures 4** and **5**), and thus on cylindrical woody branches. Experiments with a PVC rod as model system, with well-known properties and simple geometry, are therefore helpful to investigate the principles of wave propagation in branches.

From the surface displacements on both sides of the PVC rod (**Figures 3A,B**), the fundamental symmetric mode (S0) and fundamental anti-symmetric (A0) mode with lower frequency were clearly discernible in the waveforms. The fundamental wave modes are the modes with the lowest frequency of an infinite series of symmetric and anti-symmetric modes that can be stimulated in a waveguide. The absence of higher modes, which contain higher frequencies, is explained by the nature of the PLB source. Since the PLB signal typically has the highest amplitude at low frequencies (Breckenridge et al., 1990), the higher modes are not stimulated. A third type of wave mode that could exist in a cylindrical rod is the torsional mode (Bischoff et al., 2014). According to theory (Rose, 2004), torsional modes consist of displacements in the tangential direction. Because we can only observe radial displacements (normal to the rod surface) with the point-contact sensor, the torsional guided wave modes are not detected by our sensor system. To investigate the torsional guided wave modes, a wafer-type AE sensor could be used (Giurgiutiu, 2008), which is sensitive to in-plane waves.

By varying the angle between source and sensor, we shed light on the behavior of the symmetric and anti-symmetric wave modes in a cylindrical branch. The amplitude of the anti-symmetric wave mode observed normal to the rod surface decreased according to the cosine of the angle between the radial line through the sensor and the radial line through the source (**Figures 4A and 5A**). As a consequence, the A0-mode was not observable when the angle between source and sensor was 90°. When the wave propagates down the rod in this mode, the rod is put in localized oscillating bending (as schematically shown in **Figure 3C**) in the plane defined by the source location and the rod axis. The amplitude of the symmetric mode (S0-mode), in contrast, is equal in all directions (**Figures 4A and 5A**) and independent of source-to-sensor angle (**Figures 4B and 5B**). In the symmetric mode, the rod is subjected to sequential symmetric expansion and contraction of the cross sections (**Figure 3C**).

When changing the depth of the source below the branch surface, the balance between A- and S-mode changed. The amplitude of the A0-mode increased linearly with increasing distance between the source location and the rod axis (**Figures 4B and 5B**), whereas the S0-mode was not affected. The more asymmetric the source location (further from the rod axis), the more the anti-symmetric wave mode prevails. As the two modes have distinctly different frequencies (**Figure 3C**), the source location also influences the frequency content of the detected signals. Thus, signals from a particular source mechanism will show some variation in frequency features due to variation in their distance from the rod axis as well as changes in the angle between the source location and the sensor.

### Deciphering Drying-Induced AE Signals

The direct pressure wave caused by an abrupt AE source (**Figure 2B**) is clearly discernible from the far-field signal with guided wave modes (**Figure 3**). Based on the gained insights in wave propagation, we were able to distinguish near- and far-field signals in dehydrating branches. The signals depicted in **Figure 2D** were caused by nearby AE sources. As we observe the waves very close to the source, without superimposed reflections from the rod surface, we detect the direct bulk compressional waves. The nearby direct bulk waves will be similar for AE sources with similar source functions. Therefore, similarities between PLB signals (**Figure 2B**) and near-field drying-induced AE signals indicate the presence of similar AE source dynamics. Given the step-like force function of a PLB, the drying-induced signals in **Figure 2D** were most likely also caused by a sudden displacement in the close surrounding of the sensor. In the AE signals from the far field (**Figure 6**), we could distinguish a separated symmetric and anti-symmetric mode in the signals from two opposite sensors. Although these longer signals may evoke the impression that the underlying AE sources were of oscillating nature, we know that the elongated shape is caused by wave dispersion. These results suggest that displacements during bubble growth, rather than superimposed small bubble oscillations of high frequency (Vincent et al., 2014), may be a source of AE signals.

A large portion of the signals detected in the dehydrating branches fell in between both categories of signals that we have discussed, showing mixed characteristics. These AE signals may have resulted from a relatively close AE source from which the wave modes were not yet fully developed. Because of the difference in wave mode velocity, both modes are better separated when the propagation distance is larger, so that we can clearly distinguish them in the signals at a certain distance from the source (**Figure 6**). When the AE source mechanism was similar, we could extract some information on the source location. For example, based on the smaller time lag between the arrival times of both wave modes in **Figure 6A**, we could deduce that this AE event took place closer to the sensor pair than the event in **Figure 6B**. We also observe a higher amplitude ratio between A- and S-mode in **Figures 6A compared to 6B** This might indicate that the AE source in **Figure 6A** was located closer toward the surface of the branch, or that the angle between source and sensor was larger in **Figure 6B**.

However, different AE sources may stimulate the S- and A-modes with different degree. It has been shown that different source rise time (time period in which the displacement takes place in a microscopic volume) can stimulate the S- and A-modes with different degree, due to activation of different frequencies (Hamstad, 2010). Moreover, different orientations of the major displacements at the AE source (source geometry) can change the balance between S0- and A0-mode (Downs et al., 2003). In other words, distinction between the different modes may help to identify different source types (with certain source excitation time and source geometry) when the source location is constant, and vice versa.

### Challenges for the AE Technique in Plant Science

Because wave propagation along the branch has a large effect on the observed waveform characteristics, it is essential to understand the principles of guided wave modes in order to make any progress in signal interpretation. We focused our study on wave propagation in young branches. Being extremities of the hydraulic pathway, young branches are more prone to drought stress than the trunk and larger branches (McCulloh et al., 2014). Moreover, compared to leaves and petioles, measurements on branches can continue after wilting of the leaves. Branches are thus convenient study objects in terms of drought stress. However, when measuring AE signals on other plant organs, guided wave modes may be involved too. Note that the frequencies of the guided waves are inversely proportional to the lateral dimensions of the waveguide (Rose, 2004). Both leaf petioles and large trunks will act as a cylindrical waveguide, but the frequencies of resulting guided waves will be much higher or lower, respectively, than those in young branches. Leaves, having a plate-like geometry, will also guide typical symmetric and anti-symmetric wave modes. The behavior of guided wave modes in plates, called Lamb modes, has been extensively studied in the context of structural health monitoring (Hamstad, 2010; Sause et al., 2013; Park et al., 2014). When aimed at signal analysis of other plant parts, with different geometry, the behavior of guided waves will thus need to be considered too.

To make advances in AE research on plants, it is essential to choose appropriate AE equipment. Sensors should be sensitive to surface displacement in a broad frequency range in order to obtain results that can be compared to calculated waveforms. The point-contact sensor used in this study is very appropriate for this purpose, as it has a very flat spectral response over a broad frequency range (20–1000 kHz). Moreover, we switched from parameter-based to signal-based AE analysis. Instead of focusing on a few waveform features (Rosner, 2012; Wolkerstorfer et al., 2012; Vergeynst et al., 2015), we considered the whole waveform of the detected signal. Currently, high performance acquisition systems are available that are able to record and store waveforms from multiple channels at high sampling frequencies. This allows post-processing of the data and thorough analysis with an unlimited number of signal features.

The combination of state-of-the-art AE measurement techniques and finite-element modeling of wave propagation is essentially new in plant science, and could be the first step toward revealing the secrets behind AE signals in plants. Dynamic finite-element modeling is increasingly used in modern AE work to simulate the AE sources and the subsequent propagation of the displacement waves (Hamstad, 2007; Aggelis and Matikas, 2012; Sause et al., 2013; Ge et al., 2014). When the geometry and elastic properties of the considered plant part are known, the AE signal that results from a certain AE source type can be simulated at the detector location using FEM (Sause and Richler, 2015). The combination with the well-known PLB is of great value since all source characteristics are known and pseudo sensors (model simulations) are perfect point contact sensors. As this approach has proven successful in the field of material science (Sause et al., 2012b) to distinguish between different types of AE sources, AE signal analysis may provide a promising avenue also in the field of plant science.

The ability to use the wider frequency information of this new approach to identify AE sources in experiments where multiple source types are present is a significant advancement. Thorough understanding of the AE signals may lead to the identification of the underlying sources. This may greatly improve the reliability of the AE measurements for the detection of drought-induced cavitation (Vergeynst et al., 2015), which will facilitate research into drought responses of plants, our ultimate goal. Especially the identification of cavitation-induced AE signals would make this method very suitable for non-destructive and automated detection of gas emboli formation in the xylem under drought. These measurements could deliver valuable information for forest management, irrigation strategies and selection of plants for breeding under water-limited conditions.

## Conclusion

In this work we investigated how guided wave modes are developed in rod-like branches. These insights made us realize that the drought-induced AE signals probably originate from sudden abrupt AE sources, rather than oscillating sources. We introduced a new framework for deciphering AE signals from plants based on broadband point-contact sensors, high sampling rate signal recording, time-frequency analysis and FEM. More detailed AE source models could be developed to approach the actual microstructure of AE sources in plants. We believe that signal-based AE analysis supported by FEM could lead to a breakthrough in the current controversy about the actual sources of AE signals in drought-stressed plants.

## Conflict of Interest Statement

The authors declare that the research was conducted in the absence of any commercial or financial relationships that could be construed as a potential conflict of interest.
